# Dynamic Study on Synergy Mechanism and Characteristics of Particle Removal in Electrostatic Atomization

**DOI:** 10.3390/molecules30122609

**Published:** 2025-06-16

**Authors:** Chenzi Teng, Yun Zhang, Sida Ren, Jianyu Cai

**Affiliations:** 1 Institute of Urban System Engineering, Beijing Academy of Science and Technology, Beijing 100089, China; 2 Institute of Energy and Environmental Protection, Academy of Agricultural Planning & Engineering, Ministry of Agriculture and Rural Affairs, Chaoyang District, Beijing 100125, China; yuncutie@163.com; 3 Research Institute of Technology of Shougang Group Co., Ltd., Shijingshan District, Beijing 100043, China; rensida628@163.com; 4 National Engineering Research Center of Urban Environmental Pollution Control, Beijing Municipal Research Institute of Eco-Environmental Protection, Xicheng District, Beijing 100037, China; cai1123806186@163.com

**Keywords:** mechanism for enhancing efficiency of removal, gas ionization mode, ion wind effect, analysis of particle force, agglomeration and coalescence effect, stability of particle removal

## Abstract

A laboratory-scale wire plate wet electrostatic precipitator was designed and constructed to investigate the particle enhancement and capture characteristics of electrostatically charged droplets in continuous atomization mode. A comparison was made between different types of wet electrostatic precipitation mechanisms for particle removal, and the change mechanism of gas ionization mode under the action of charged droplets was analyzed. Experimental investigations were conducted on the effects of electrospray on corona discharge “ionic wind”, as well as the force mechanism, agglomeration effect, and removal stability of particles under the synergistic action of electrostatic atomization and an electric field. The results demonstrated that electrospray mode could enhance the interaction between droplets and particles, promote the coagulation and accumulation of fine particles, increase their diameter to larger sizes that are easier to capture, and achieve high particle collection efficiency with significantly reduced water consumption while maintaining high corona current and particle capture effectiveness.

## 1. Introduction

According to the Global Burden of Disease study, air pollution is responsible for an estimated annual global mortality rate of up to 7 million deaths [1]. Among these fatalities, over 2.1 million premature deaths are attributed to the increased concentration of suspended particulate matter such as PM2.5 in the atmosphere. The adverse impacts of fine particulate matter on both the environment and human health have received significant attention. Reducing emissions serves as a primary driving force for improving air quality globally. Wet electrostatic precipitation technology has been extensively studied due to its effectiveness in capturing fine particles while mitigating issues such as back corona and particle secondary reflux; however, it commonly faces challenges including high water consumption, potential secondary emission pollution, and increased operational costs [2,3].

Extensive research has been conducted in the field of non-equilibrium plasma to improve particle removal efficiency. Previous studies [4,5,6,7,8] have examined variations in ion mobility and corona onset voltage and current, and they have also elucidated the mechanisms and impact of factors such as flue gas temperature [9], composition [10], humidity [11], other operational parameters, and polarization parameters [12] on corona discharge characteristics. Additionally, spatial ion density prediction models have been established, along with a correction model for corona onset voltage. Furthermore, research has primarily focused on investigating the effects of cleaning water film [13,14,15], improving the distribution of cleaning water film [16,17], flue gas humidification techniques [18,19], mechanical spray methods [20,21], and multifunctional synergistic removal approaches [22,23] to understand the effect of wet electrostatic precipitation on particle capture efficiency. Moreover, a systematic disclosure of comprehensive insights into particle agglomeration characteristics during voltage variation processes within specific dust removal areas under different flue gas temperatures and dust concentrations has been provided.

In the investigation of electrostatic aerosolization enhancement for capturing fine particulate matter, the formation mechanism and influencing factors of electrostatic aerosolization have been explored. This has resulted in quantitative correlations among parameters such as corona current, droplet diameter, charge-to-mass ratio, and droplet flow rate [24,25,26,27,28]. It was observed that the charge-to-mass ratio of the generated charged droplets could reach 50% of the critical charge-to-mass ratio with a diameter only 40–50% of conventional mechanical sprays. Additionally, it was noted that the limiting charge of the droplets would decrease during evaporation and secondary aerosolization might occur [29]. Regarding improvements in fine particulate matter removal efficiency through electrostatic aerosolization, it was discovered that the velocity of charged droplets formed by electrostatic fragmentation aerosolization could reach up to 30 m/s [30], thereby enhancing particle charging effects as well as collision, interception, coagulation, and electrostatic adsorption processes. This approach demonstrates clear advantages in terms of enhancing capture efficiency and reducing water consumption [31,32,33,34]. However, further research is needed to investigate the mechanism and characteristics of fine particulate matter enhancement through cooperation between an electrostatic field and charged liquid droplets for continuous mist generation known as the electrohydrodynamic atomization mode.

## 2. Methods and Materials

### 2.1. Experimental System

The objective of this article is to introduce an efficient, low-energy, and environmentally friendly method for controlling fine particulate matter. In this study, a laboratory-scale wet electrostatic precipitator was designed and constructed to employ an electrostatic field for simultaneously charging droplets and promoting continuous atomization. The experimental setup consists of a pollutant generation system, a high-voltage power supply system, a charged droplet synergistic electrostatic field dust removal system, and a measurement and analysis system. The experiment was conducted at room temperature (25 °C). At the terminal end of the experimental system, a flue gas fan was used to maintain a negative pressure condition, thus preventing the escape of flue gas.

**Figure 1:**
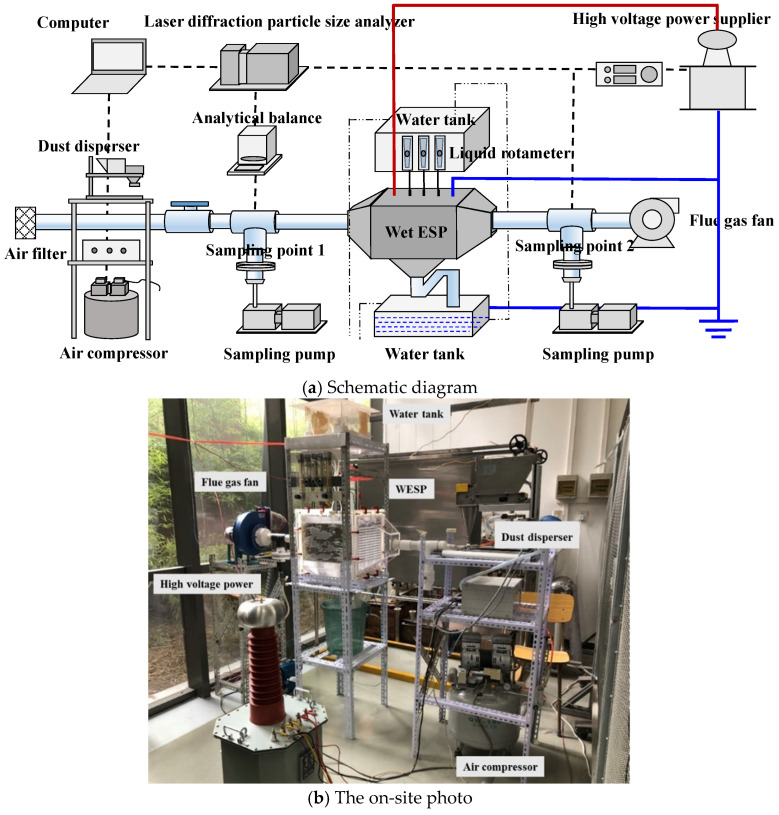
The experimental system.

The overall process of the experimental system is as follows: particles and air combine to form a dust-laden airflow through the pollutant generation system to simulate flue gas generation; after passing through the measurement and analysis system, the flue gas enters the charged droplet synergistic electrostatic field dust removal system, where efficient particle removal is achieved under the influence of the high-voltage power supply system. Finally, the purified flue gas is discharged into the atmosphere via an induced draft fan with comprehensive measurement and analysis conducted throughout (as depicted in Figure 1).

### 2.2. Steps and Methods

In the experimental setup, the residence time of dust-laden airflow and the smoke environment can be regulated by adjusting the high-voltage power supply. Meanwhile, the electric field intensity is determined by experimental parameters such as the corona line and plate spacing. Assuming an airtight structure without any leakage and equal inlet–outlet air volumes in the precipitator, Equation (1) allows for the calculation of the total efficiency *η* using measurement results obtained from the sampling and measurement system.

(1) $$\eta =(1-M_{2}/M_{1})\times 100\%$$

In Equation (1), M_1_ and M_2_ represent the masses of particles at the inlet and outlet of the precipitator, respectively (in grams). The classification efficiency refers to the removal efficiency of particles in each size segment, which can be determined by using Equation (2).

(2) $$\eta_{i}=(1-M_{2i}/M_{1i})\times 100\%$$

In Equation (2), M_1i_ and M_2i_ represent the mass (g) of particles with a particle size of dp_i_ at the inlet and outlet of the precipitator, respectively. The frequency distribution of particles with a particle size of dp_i_ at the inlet and outlet of the precipitator is expressed by Equations (3) and (4), respectively.

(3) $$f_{i}=M_{1i}/\sum_{i=1}^{n}M_{1i}=M_{1i}/M_{1}$$

(4) $$g_{i}=M_{2i}/\sum_{i=1}^{n}M_{2i}=M_{2i}/M_{2}$$

By substituting Equations (3) and (4) into Equation (2), the classification efficiency can be expressed as follows:

(5) $$\eta_{i}=(1-\frac{M_{2}\cdot g_{i}}{M_{1}\cdot f_{i}})\times 100\%=[1-(1-\eta )\cdot \frac{g_{i}}{f_{i}}]\times 100\%$$

The values of g_i_ and f_i_ in Equation (5) are obtained through laser particle size analysis, while the total efficiency *η* is calculated by evaluating the mass of particles sampled and gas flow before and after precipitator treatment. By utilizing Equation (5), the penetration rate for a specific particle size segment dp_i_ can be computed.

(6) $$P_{i}=1-\eta_{i}=(1-\eta )\cdot \frac{g_{i}}{f_{i}}\times 100\%$$

**Figure 2:**
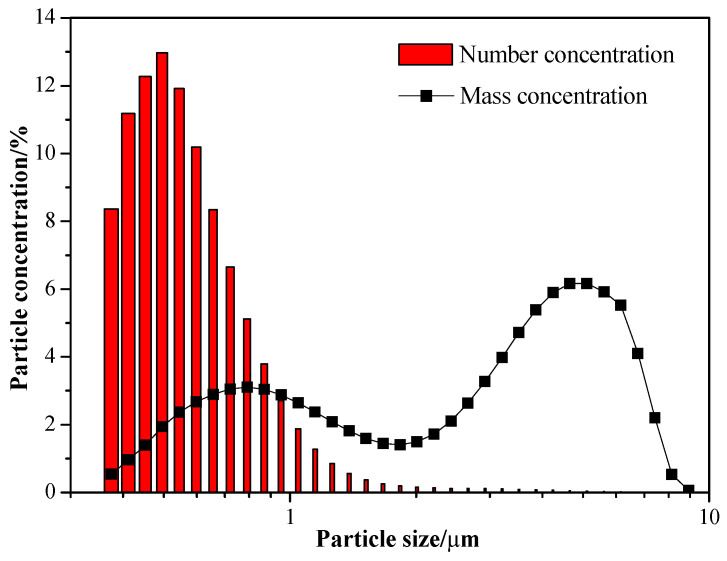
Concentration distributions of talcum powder particles in the experiment.

Talcum powder was selected as the target pollutant in the experiment due to its small particle size, high electrical resistance, and water-repellent properties. Figure 2 illustrates the distribution of talcum powder particle sizes, indicating that the mass proportions of particles with particle sizes below 1 μm, between 1 μm and 2.5 μm, and above 2.5 μm are 24.82%, 18.64%, and 56.54%, respectively.

## 3. Results and Discussion

### 3.1. Mechanisms of Particle Removal in Different Types of Wet Electrostatic Precipitators

**Figure 3:**
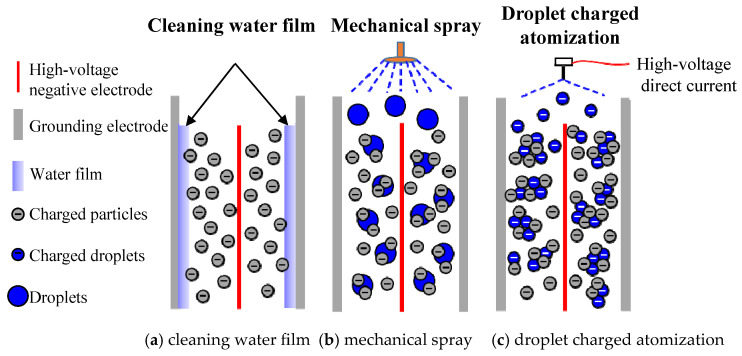
Comparison of particle removal mechanisms of wet electrostatic precipitators.

The application methods of water in wet electrostatic precipitators exhibit significant variations in their impact on particle capture efficiency and operational water consumption. Figure 3 illustrates the contrasting particle removal mechanisms employed by wet electrostatic precipitators utilizing cleaning water film, mechanical spray, and droplet charged atomization. Table 1 presents a comparison of the key parameters of various types of wet electrostatic precipitators.

The dust removal water film in the traditional wet electrostatic precipitator, as illustrated in Figure 3a, primarily serves to eliminate captured dust particles on the electrode plate. The uniform distribution of this water film is a crucial factor that influences capture efficiency. Non-uniform distribution can lead to trenches and dry spots, significantly diminishing the enhancement effect of water on corona discharge and particle removal within the electrostatic field of the wet electrostatic precipitator; however, achieving an evenly distributed water film typically requires a substantial amount of water.

The improved mechanical spraying method, as depicted in Figure 3b, offers dual advantages. Firstly, the presence of droplets within an electrostatic field facilitates ion migration and collision ionization, thereby enhancing corona current. Secondly, a high concentration of fine mist droplets promotes collisions between droplets and particles within the capture area, effectively facilitating condensation and agglomeration among fine particles while simultaneously reducing water consumption.

The wet electrostatic precipitator, illustrated in Figure 3c, utilizes droplet charged atomization to achieve enhanced droplet atomization through the synergistic effect of high-voltage electric fields. As a result, smaller charged droplets are generated and propelled towards the electrode plate at an accelerated velocity, leading to improved interaction between droplets and particles that promotes the agglomeration and accumulation of fine particles. This process results in their enlargement into larger easily capturable particles, thereby achieving higher particle capture efficiency while significantly reducing water consumption.

### 3.2. Mechanisms of Gas Ionization in Presence of Charged Atomized Droplets

The influence of corona discharge on particle charging and capture efficiency plays a crucial role in electrostatic precipitators. The utilization of negative corona discharge, which is commonly practiced in electrostatic dust removal systems, generates an abundance of negative ions at the discharge electrode within a high-voltage electric field. Dust particles acquire charge through collisions and effectively migrate towards the collection plate under the influence of electric field forces to ensure efficient capture.

**Figure 4:**
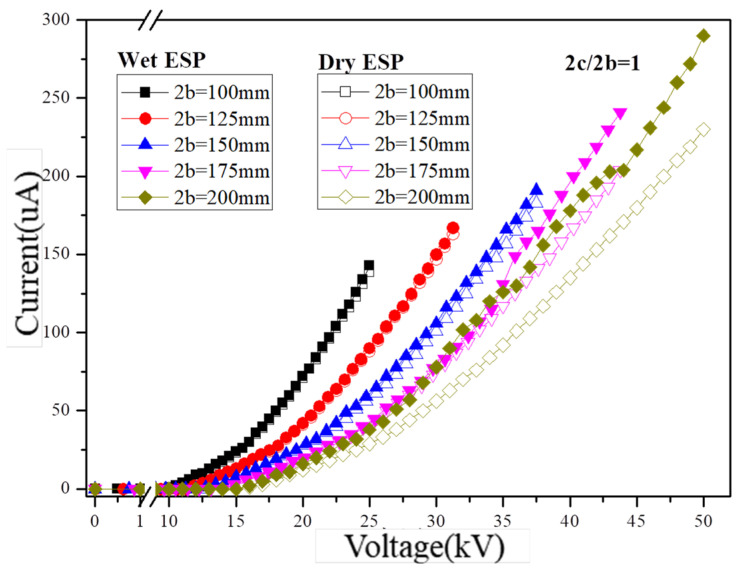
Current–voltage characteristics in the dry- and wet-type ESPs (2c/2b = 1).

The research demonstrates that an increased corona current density positively affects particle charging and enhances particle capture. Figure 4 presents the analysis of the volt–ampere characteristics of the dust collector under various operating voltages. In this study, a single channel layout was employed for the dust removal device with an electric field strength ranging from 0 to 5 kV/cm and five plate spacings at intervals of 100 mm, 125 mm, 150 mm, 175 mm, and 200 mm, respectively. Correspondingly, the operating voltages at a fixed electric field strength of 5 kV/cm were set as follows: 25.0 kV, 31.3 kV, 37.5 kV, 43.8 kV, and 50.0 kV, respectively. Each working condition includes two corona wires and one nozzle, with the flow rate of each nozzle set to 20 mL/min. The spacing between corona wires is consistent with the plate spacing.

The corona current demonstrates an increasing trend as the operating voltage exceeds the corona onset voltage under various experimental conditions, as depicted in Figure 4. Additionally, a negative correlation is observed between the corona current and plate spacing at a given voltage. Moreover, both the operating voltage and plate spacing positively contribute to the generation of corona current induced by charged droplets. The electric field strength varied from 2 kV/cm to 5 kV/cm, resulting in a corresponding increase in the dry condition corona current from 12 μA to 230 μA for a plate spacing of 200 mm. Furthermore, the enhancement effect of droplet charged atomization on the corona current increased from 2 μA to 60 μA. Under droplet charged atomization conditions, increases were observed in respective corona currents at five different plate spacings (143 μA, 167 μA, 191 μA, 241 μA, and 290 μA) as the electric field strength increased to 5 kV/cm. Additionally, with an increase in plate spacing from 100 mm to 200 mm, the contribution of charged droplets and the electrostatic field to the overall corona current increased from 2.9% to 26.1%.

**Figure 5:**
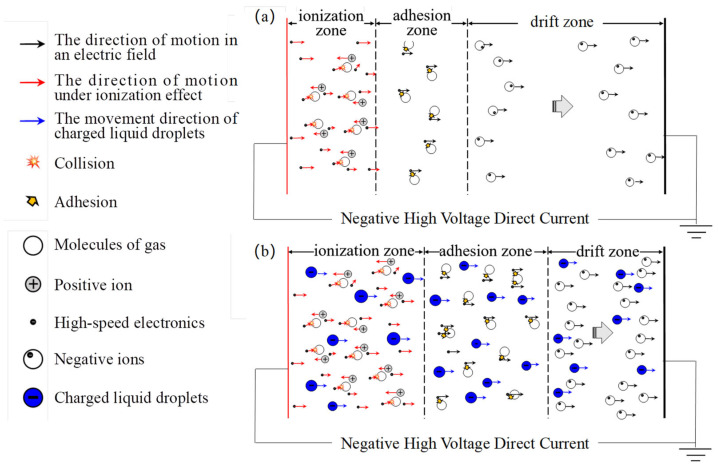
Discharge modes of gas ionization as a result of charged droplets. (**a**) conventional corona discharge. (**b**) corona discharge as a result of the influence of charged droplets.

The corona discharge is typically divided into three zones, namely the ionization zone, adhesion zone, and drift zone. Figure 5 illustrates the mechanism of corona discharge in these zones under the influence of charged droplets. It is evident that within the ionization zone, a strong electric field is generated in close proximity to the discharge electrode, facilitating gas molecule ionization and resulting in a significant abundance of free electrons. These high-velocity electrons continuously collide with gas molecules, leading to the formation of electron–cation pairs through ionization. The newly formed electrons possess enhanced capacity and persistently interact with surrounding gas molecules under the influence of the electric field, inducing their ionization and consequently initiating an electron avalanche.

In the adhesion zone, a significant increase in the density of negative ions is observed due to the collision and adsorption of a large number of electrons from the ionization zone onto gas molecules as a result of the influence of charged droplets. Consequently, numerous negative ions and charged droplets migrate from the adhesion zone to the drift zone. If their concentration exceeds saturation levels for negative ions, this region generates free electrons and molecules. Subsequently, driven by an electric field force, numerous negative ions and free electrons move towards the plate in the drift zone, thereby enhancing current formation through conventional corona discharge.

### 3.3. Influence of Electrospray on “Ionic Wind” in Corona Discharge

The findings from previous studies indicate that when Ehd is greater than or equal to Re2 (Ehd ≥ Re2), the secondary flow phenomenon caused by the current body exerts a substantial impact on the flow field inside the dust collector, leading to the formation of unstable turbulence in the fluid [35,36]. Therefore, the similarity constant Ehd/Re2 is used to evaluate the potential impact of secondary flow induced by the current body on both dry and wet conditions of the filter’s flow field. Subsequently, an analysis is conducted to investigate how charged droplets in an electrostatic field affect ion wind within the filter. Notably, in accordance with international standards for current bodies [37], non-dimensional parameters Ehd and Re are employed as representations of system numbers and Reynolds numbers, respectively. The system number Ehd serves as a criterion for evaluating ion wind intensity within the filter, while the Reynolds number Re characterizes current body flow. Equations (7) and (8) are used for calculating these non-dimensional parameters.

(7) $$E_{hd}=\frac{I_{0}\cdot L^{3}}{\rho_{f}\cdot v_{f}^{2}\cdot \mu_{i}\cdot A}$$

(8) $$Re=\frac{L\cdot U_{0}}{v_{f}}$$

The current (A) is referred to as I_0_ in Equations (7) and (8), while the characteristic length (m) is denoted by L. Fluid density (kg/m^3^) is symbolized as ρf, fluid kinematic viscosity (Pa·s) is denoted as v_f_, and ion mobility (m^2^/s/V) is indicated by μ_i_. A represents the total area of the dust collector in square meters, while U0 signifies fluid velocity in meters per second. In this study, v_f_, ρ_f_, and μ_i_ are set to 1.81 × 10^–5^ Pa·s, 1.29 kg/m^3^, and 2 × 10^–4^ m^2^/s/V, respectively. The variation law of Ehd ≥ Re^2^ under different working conditions with the corona diameter serving as the characteristic length is illustrated in Figure 2 and Figure 3. It should be noted that the numerical value for the corona diameter equals twice the thickness t_h_ [38], which can be calculated using Equation (9).

(9) $$t_{h}=1.56\cdot r_{w}^{0.65}$$

**Figure 6:**
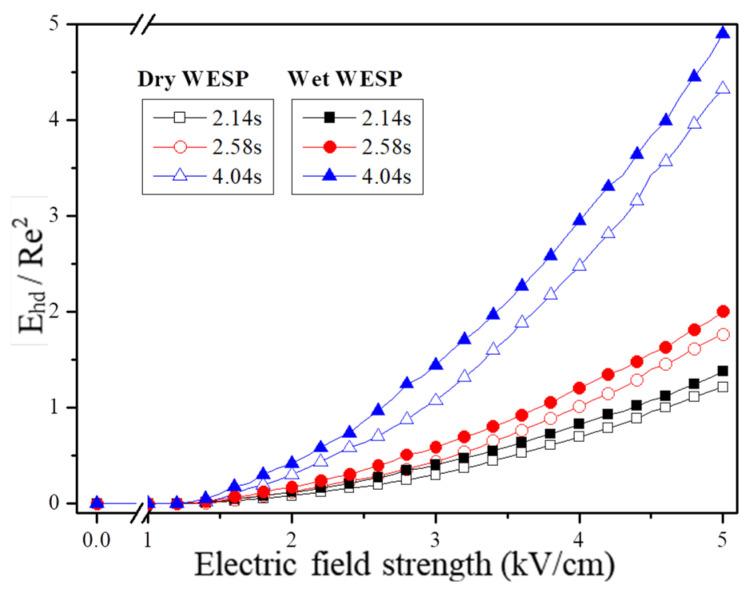
Variation in E_hd_ ≥ R_e_^2^ in ESPs and WESPs.

According to Equation (9), the thickness of the corona discharge region is determined by the curvature radius r_w_ of the discharge electrode. The corona wire used in this experiment has a curvature radius of 0.5 mm. Figure 6 illustrates the variation in E_hd_ ≥ R_e_^2^ for dry and wet electrostatic precipitators. At staying times of 2.14 s, 2.58 s, and 4.04 s, the influence of secondary flow generated by current on the flow field increases with both increasing electric field strength and staying time. Furthermore, it is observed that the impact of ion wind generated by corona discharge consistently exceeds that in dry electrostatic precipitators.

This phenomenon is attributed to the generation of space charge through corona discharge, which subsequently induces an electric volume force that acts on the surrounding air under the influence of an electrostatic field. This results in an airflow effect and ion wind generation. As the strength of the electric field and residence time increase, both charge and ion density within the electric field space escalate, leading to a rise in the E_hd_ current system number and similarity constant (E_hd_ ≥ R_e_^2^), consequently amplifying the impact of corona discharge-induced ion wind on the flow field.

In the wet state, when the surface charge of charged droplets exceeds the Rayleigh limit, they undergo breakup and atomization under the influence of an electric field before moving towards the electrode. This phenomenon has dual effects: firstly, it increases air humidity due to charged droplets, thereby promoting an increase in current I_0_ and resulting in larger values for both the current coefficient E_hd_ and similarity coefficient E_hd_ ≥ R_e_^2^. Secondly, this process enhances the surrounding airflow around the droplets, further amplifying the impact of current-induced secondary flow on the internal flow field of electrostatic precipitators. Consequently, within an electric field, charged droplets enhance the ion wind effect.

From Figure 6, it is evident that when the electric field strength of the wet electrostatic precipitator exceeds 4.4 kV/cm, 3.8 kV/cm, and 2.6 kV/cm, respectively, the similarity constant E_hd_/R_e_^2^ ≥ 1 indicates a relatively pronounced secondary flow induced by the current fluid compared to the main flow in the wet electrostatic precipitator. Additionally, under an electric field strength of 4.6 kV/cm, 4.0 kV/cm, and 3.0 kV/cm, respectively, for dry electrostatic precipitators, there is a reduction in the starting electric field strength by approximately 0.2–0.4 kV/cm for a significant appearance of secondary flow during a liquid droplet charging mistification action taking place. The ion wind generated by charged liquid droplets significantly influences the internal flow field of the precipitator and further enhances corona discharge-induced ion wind generation; thus, incorporating charged droplets at equivalent electric field strengths can enhance particle driving speed and facilitate particle capture.

### 3.4. Motive Force Mechanism of Particles Influenced by Charged Droplets

The forces acting on particles within the precipitator are highly complex, with the significance and magnitude of various forces depending on the type of precipitator and particle location [39]. The mechanical analysis of particulate matter is essential for studying its motion characteristics [40,41]. In a wet electrostatic precipitator, there is an intricate system involving interactions among gas flow, dust particles, and charged droplets. Due to their rapid formation within an electrostatic field where they acquire high charge-to-mass ratios and small sizes comparable to solid particles in terms of force analysis, this entire motion system can be simplified into a two-phase flow comprising gas and solid particles.

In gas–solid two-phase flow, the solid experiences three primary forces, namely field force, fluid force, and solid force. The field force refers to the external physical fields acting on particles. Within an electrostatic precipitator, the field force includes gravity, external electric fields, electric fields within the dust layer, thermophoretic forces, concentration gradient forces, surface tension, and more. The fluid forces exerted on solid particles in the entire system primarily include buoyancy, drag force, Basset force, additional mass force, Saffman force, Magnus force, and pressure gradient force. Conversely, the solid forces acting upon solid particles in distinct regions predominantly involve van der Waals force, collision force, liquid bridge force, electrostatic attraction and repulsion.

The movement space of particles in the precipitator is divided into three regions based on their motion characteristics in the electric field, including the capture region (δ < Δd ≤ b), the capture layer (0 < Δd ≤ δ), and finally against or near walls where no displacement occurs (Δd = 0). This division allows for an analysis of the force types acting on solid particles in two-phase flow. Here, Δd represents the distance between particles and the surface of the electrode plate, b represents the heteropole distance, and δ represents capture layer thickness. It is assumed that (1) dust particles in the airflow are fully dispersed; (2) airflow velocity in the core area of airflow turbulence is uniform; (3) the water film formed on the wet ESP’s electrode plate surface is uniformly distributed; (4) particles can be completely removed by the water film after reaching the surface of the electrode plate; and (5) there is no interference from other factors such as particle reflux and anti-corona. Based on these assumptions, a comparison is made between dry and wet ESPs regarding types of forces applied to particles, and an analysis of the particle force mechanism is presented in Table 2.

In the collection zone, due to the complete dispersion of dust particles, it falls under the category of dilute phase flow, where the repulsive force between particles can be disregarded. Moreover, in dry electrostatic precipitators and traditional wet electrostatic precipitators with mechanical vibration or water spray cleaning, there are negligible variations in temperature and velocity within the collection zone, enabling an approximation of an isothermal and uniform field. However, in electrostatic precipitators employing mechanical spraying and charged droplets, considerations must be given to pressure gradient (F_p_), Saffman lift force (F_s_), and Magnus force (F_M_). Furthermore, in wet (charged droplet) electrostatic precipitators, static charge-induced attraction/repulsion forces between particles (F_i_/F_j_) must also be taken into account.

Under the influence of an electrostatic field, the particles undergo significant changes in velocity and temperature gradient as they move towards the collection layer. Therefore, when considering a wet electrostatic precipitator with a cleaning water film, it is crucial to take into account additional forces such as the Basset force F_Ba_, mass force F_vm_, Saffman lift F_s_, Magnus force, liquid bridge force F_y_, and concentration gradient force Fc resulting from the evaporation of the cleaning water film.

When particles reach the wall and are captured (Δd = 0), they experience both the electric field force Fm and van der Waals force F_v_ within the dust layer on the plate surface in dry electrostatic precipitators. Additionally, there is an interaction of electrostatic force F_j_/F_i_. Contrarily, wet electrostatic precipitators remove particles by using a water film when they reach the plate surface. This eliminates other fluid and solid forces while creating a surface tension F_z_ between particles and the liquid film. In comparison with water film cleaning and mechanical spraying, the utilization of charged droplet atomization in wet electrostatic precipitators enhances the interaction force between charged droplets and particles within the capture area. Therefore, it facilitates particle coalescence and promotes the efficient capture of fine particles.

### 3.5. Synergistic Effect of Charged Droplet Electrostatic Atomization and Electric Field on Particle Aggregation

**Figure 7:**
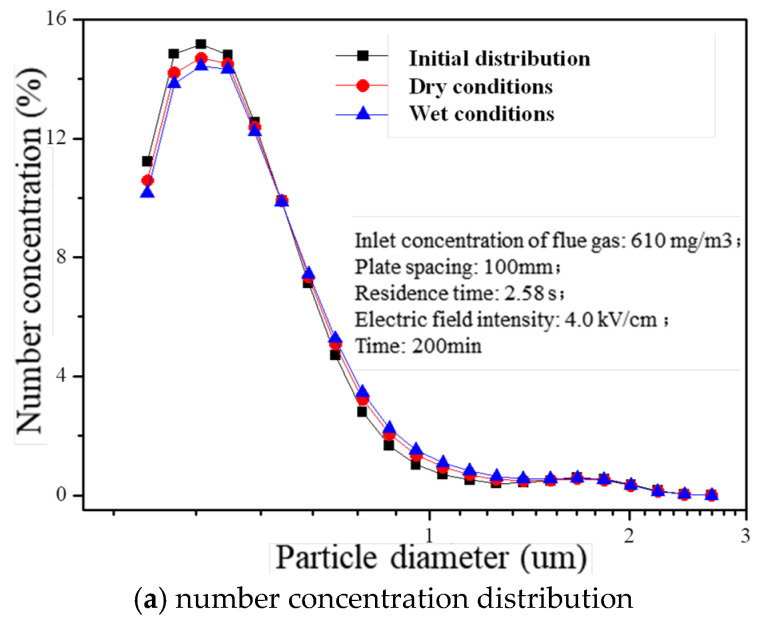

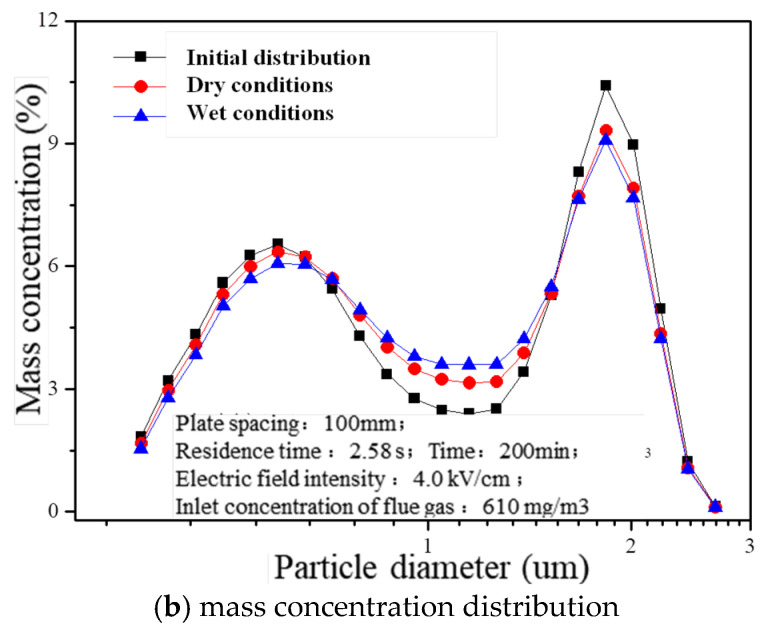
Concentration distribution of particles with different treatment processes.

The residence time (2.58 s), electric field intensity (4.0 kV/cm), and inlet concentration of flue gas (610 mg/m^3^) were precisely controlled to investigate the impact of charged atomization droplets on fine particle agglomeration. After operating a laboratory-scale wet electrostatic precipitator continuously for 200 min, noticeable changes were observed in the concentration distribution across various size ranges within treated flue gas, as depicted in Figure 7. Compared with the initial distribution under dry conditions, the presence of charged liquid droplets led to reductions by 11.8% and 6.0%, respectively, regarding PM0.5 number concentration within outlet flue gas. Additionally, there was an increase in particle diameter corresponding to D90 from its initial value of 0.809 μm to values of 0.823 μm and 0.846 μm under both dry and wet conditions, respectively. This suggests that certain fine particles tend to aggregate into larger particles due to the influence of charged mist droplets, resulting in an overall increase in particle size within the flue gas.

**Figure 8:**
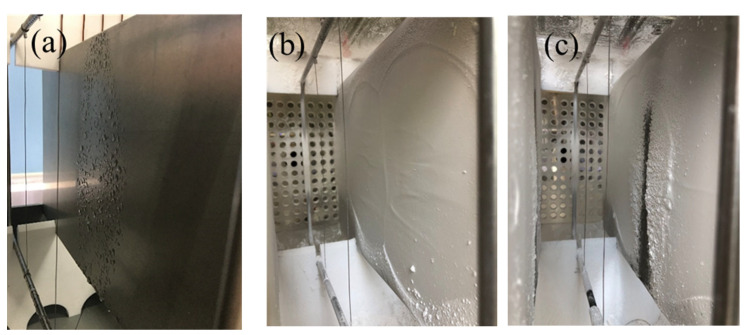
Effect of particle deposition on the surface of dust collector plate. (**a**) no particle conditions. (**b**) dry conditions. (**c**) wet conditions.

The deposition effect of particles on the surface of the dust collector plate reveals that the charged droplets interact synergistically with the electrostatic field, resulting in a uniform formation of water mist on the plate’s surface (Figure 8a). This mist effectively wets the plate, as shown in Figure 8c, where it is evident that more dust is captured by the wetted plate covered with charged water mist compared to other areas. Furthermore, there is a significant increase in dust layer thickness observed on its surface when compared to dry conditions, as depicted in Figure 8b. On the one hand, this enhanced interaction between charged droplets and fine particles within an electrostatic field promotes their condensation and growth into larger sizes, facilitating their capture. On the other hand, these charged droplets elevate flue gas humidity levels and reduce the specific resistance of dust particles to some extent, thereby aiding particle capture, as evidenced by the substantial thickening of the dust layer within this region.

### 3.6. Stability of Particle Removal Under Combined Effect of Electrostatic Atomization and Electrostatic Field

**Figure 9:**
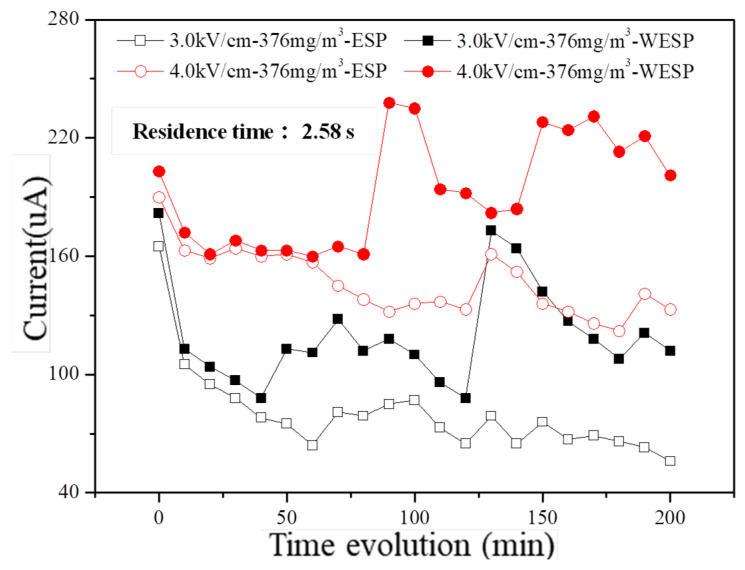
Time evolution of discharged current in laboratory conditions with different electric field intensities.

The discharge characteristics of the dust collector under different electric field strengths are depicted in Figure 9, providing further insights into the stability of fine particulate matter removal performance by charged droplets. In the dry electrostatic dust collector, the corona current gradually decreases with increasing operation time; however, in the wet electrostatic dust collector, it remains relatively stable during the initial stage of operation and slightly increases over time due to the continuous misting action by charged droplets. Moreover, consistently higher discharge currents are observed in wet conditions compared to dry conditions, and this difference is amplified with prolonged operation.

The experimental results demonstrate that the collaboration of charged droplets with an electrostatic field facilitates the sustained maintenance of a consistently high corona discharge current under long-term operational conditions compared to dry conditions. During the prolonged operation of the electrostatic precipitator, dust particles accumulate on the electrode surface due to the influence of electrostatic force, leading to the formation of an insulating dust layer. In dry conditions, this dust layer continues to accumulate and thicken on the electrode surface, thereby diminishing the electric field strength between the electrode and corona wire over time. Consequently, there is a gradual decline in corona discharge and a progressive decrease in current.

Conversely, in wet conditions, charged droplets not only increase the relative humidity within flue gas but also reduce the specific resistance of dust, thereby facilitating effective dust collection. Furthermore, these charged droplets continuously clean both electrodes and corona wires within the electric field during prolonged operation; as a result, surfaces remain clean for extended periods, which partially alleviates the inhibitory effects caused by accumulated dust layers on corona discharge performance. Consequently, corona discharge currents consistently exceed those observed in dry electrostatic precipitators while exhibiting an increasing disparity over time. Importantly, enhancing the electric field strength from 3.0 kV/cm to 4.0 kV/cm significantly improves discharge characteristics aligning with voltage’s impact on volt–ampere characteristics.

**Figure 10:**
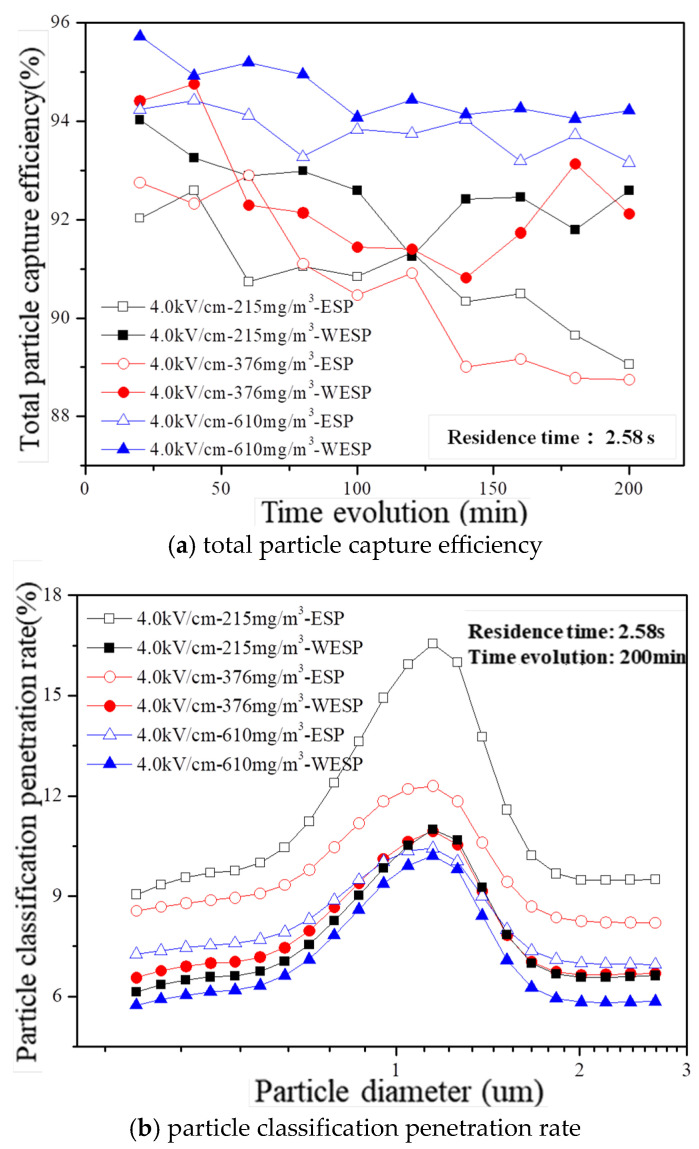
Time evolution of particle capture feature in laboratory conditions with different inlet dust concentrations.

The variation in both total particle capture efficiency and particle penetration rate over time is further demonstrated in Figure 10a,b considering an electric field intensity of 4.0 kV/cm, a residence time of 2.58 s, and dust concentrations ranging from 215 mg/m^3^ to 610 mg/m^3^. It can be observed that the wet electrostatic precipitator consistently exhibits higher capture efficiency compared to that of the dry electrostatic precipitator within a runtime span of 200 min, thus providing additional evidence for the relationship between total particle capture efficiency and the combined effect of electrically charged droplets and an electrostatic field. Furthermore, even when there is an increase in dust concentration from 215 mg/m^3^ to 610 mg/m^3^, the wet electrostatic precipitator maintains its high efficiency throughout this range. Additionally, due to the influence of electrically charged droplets, there is a decrease in the particle classification penetration rate from a range of 6.14–11.00% to a range of 5.75–10.21%.

## 4. Conclusions

In the electrodynamic atomization mode, droplet charging is combined with an electrostatic field to induce the fragmentation of charged droplets under a high-voltage electric field. This results in generating smaller-sized charged droplets after atomization that exhibit enhanced velocity towards the plate. Consequently, this mode facilitates intensified interaction between droplets and particles, promoting a further agglomeration and aggregation of fine particles while increasing their diameters for easier capture. Thus, it achieves heightened particle capture efficiency while significantly reducing water consumption and maintaining consistent levels of corona current and particle capture effectiveness during long-term operational conditions. Compared with the traditional wet electrostatic precipitation technology, the method proposed in this paper demonstrates significant advantages in enhancing particle charging, collision, interception, coagulation, and electrostatic adsorption. These improvements collectively contribute to a more efficient removal of fine particles and enhanced overall performance. The mechanism and characteristics underlying synergistic particle removal primarily are summarized as follows:

(1) Charged droplets have a significant impact on corona discharge. as they influence both the ionization and deposition zones. In the ionization zone, droplets alter the distribution of the electric field surrounding electrons, resulting in a localized enhancement of electric field strength. This amplification increases the energy levels of fast-moving electrons and their ability to ionize gas molecules. In the adhesion region, charged droplets greatly enhance negative ion formation while facilitating the movement of negative ions and free electrons towards the plate in the drift zone. This intensifies the overall current generated by conventional discharge.
(2) The phenomenon of enhanced “ion wind” is observed when droplets are charged and atomized in combination with an electrostatic field. As the electric field strength increases, the charged droplets undergo atomization, resulting in a reduction in particle size. These droplets generate high-speed gas flow as they move towards the plate, promoting accelerated air jetting between the corona area and the plate. This phenomenon exhibits characteristics that enhance both the “ion wind” effect and particulate matter propulsion speed.
(3) The co-action between electrospraying liquid droplets and an electrostatic field can enhance the interaction force between particles and charged droplets, thus affecting the movement of particles in different spatial dimensions. These forces vary significantly in terms of type and magnitude. In both the collection area and collection layer, particles mainly encounter forces from the electric field, fluid flow, and solid surfaces. On the other hand, at the wall surface, particles are primarily influenced by forces generated by the electric field.
(4) The presence of electrophoretic droplets facilitates the agglomeration and aggregation of fine particles, promoting their growth into larger particles that are more easily captured. With a flue gas retention time of 2.58 s and an electric field intensity of 4.0 kV/cm, the dust concentration increases from 215 mg/m^3^ to 610 mg/m^3^, while the penetration rate for particle classification decreases from 6.14–11.00% to 5.75–10.21%. This reduction in the penetration rate is attributed to the influence of electrophoretic droplets on particle behavior, resulting in a shift in the concentration distribution within the flue gas towards lower concentrations of fine particles and higher concentrations of coarse particles.

## Figures and Tables

**Table 1:** A comparison of the key parameters of various types of WESPs.

Water Supply	Structure of WESP	Gas Residence Time (s)	Electric Field Intensity (kV/cm)	Particle Size (μm)	Particle Removal Efficiency (%)	Liquid/Gas Ratio (L/m^3^)
Cleaning water film	wire toplate/wire totube	0.19–2.0	1.76–4.78	0.0168–300	20–99.7	0.77–5.89
Mechanical spray	wire to plate	0.74–2.5	2–3.67	0.01–10	65–100	0.32–1.3
Droplet charged atomization	wire to plate	2.14–4.04	3.0–4.0	0.37–2.66	89–96	0.09–0.17

**Table 2:** Analysis of particle force mechanism in dry and wet electrostatic precipitators.

Operational Parameters	Spatial Location of Particle	Types of Field Forces	Types of Fluid Force	Types of Solid Forces
Electric precipitator of the dry type	δ < Δd ≤ b	F_g_, F_e_	F_D_	
	0 < Δd ≤ δ	F_g_, F_e_	F_D_, F_Ba_, F_vm_, F_s_, F_M_	
	Δd = 0	F_g_, F_m_		F_v_, F_j_/F_i_
Wet electrostatic precipitator employing water film	δ < Δd ≤ b	F_g_, F_e_	F_D_	
	0 < Δd ≤ δ	F_g_, F_e_, Fth, F_c_	F_D_, F_Ba_, F_vm_, F_s_, F_M_	F_y_
	Δd = 0	F_g_, F_z_		
Wet electrostatic precipitator employing mechanical spray technology	δ < Δd ≤ b	F_g_, F_e_	F_D_, F_s_, F_M_, F_p_	F_pe_, F_y_
	0 < Δd ≤ δ	F_g_, F_e_, F_c_	F_D_, F_Ba_, F_vm_, F_s_, F_M_, F_p_	F_y_
	Δd = 0	F_g_, F_z_		
Wet electrostatic precipitator employing electrostatically charged droplet	δ < Δd ≤ b	F_g_, F_e_	F_D_, F_s_, F_M_, F_p_	F_pe_, F_y_, F_j_/F_i_
	0 < Δd ≤ δ	F_g_, F_e_, F_c_	F_D_, F_Ba_, F_vm_, F_s_, F_M_, F_p_	F_y_
	Δd = 0	F_g_, F_z_		

Notes: field forces include gravity (F_g_), external electric field force (F_e_), electric field force in the dust layer (F_m_), thermophoresis force (F_th_), concentration gradient force (F_c_), and surface tension force (F_z_). Fluid forces consist of resistance force (F_D_), Basset force (F_Ba_), additional mass force (F_vm_), Saffman lift force (F_s_), Magnus force (F_M_), and pressure gradient force (F_p_). Solid forces encompass van der Waals attraction (force F_v_), collisional interaction (force F_pe_), liquid bridge formation (force F_y_), electrostatic attraction (force F_j_) and electrostatic repulsion (force F_i_).

## Data Availability

Data are contained within the article.
